# Examining data visualization pitfalls in scientific publications

**DOI:** 10.1186/s42492-021-00092-y

**Published:** 2021-10-29

**Authors:** Vinh T Nguyen, Kwanghee Jung, Vibhuti Gupta

**Affiliations:** 1 Department of Information Technology, TNU – University of Information and Communication Technology, Thai Nguyen, Vietnam; 2 Department of Educational Psychology, Leadership, and Counseling, Texas Tech University, Lubbock, TX 79409 United States; 3 Department of Computer Science and Data Science, Meharry Medical College, Nashville, TN 37208 USA

**Keywords:** Data visualization, Graphical representations, Misinformation, Visual encodings, Association rule mining, Word cloud, Cochran’s Q test, McNemar’s test

## Abstract

Data visualization blends art and science to convey stories from data via graphical representations. Considering different problems, applications, requirements, and design goals, it is challenging to combine these two components at their full force. While the art component involves creating visually appealing and easily interpreted graphics for users, the science component requires accurate representations of a large amount of input data. With a lack of the science component, visualization cannot serve its role of creating correct representations of the actual data, thus leading to wrong perception, interpretation, and decision. It might be even worse if incorrect visual representations were intentionally produced to deceive the viewers. To address common pitfalls in graphical representations, this paper focuses on identifying and understanding the root causes of misinformation in graphical representations. We reviewed the misleading data visualization examples in the scientific publications collected from indexing databases and then projected them onto the fundamental units of visual communication such as color, shape, size, and spatial orientation. Moreover, a text mining technique was applied to extract practical insights from common visualization pitfalls. Cochran’s Q test and McNemar’s test were conducted to examine if there is any difference in the proportions of common errors among color, shape, size, and spatial orientation. The findings showed that the pie chart is the most misused graphical representation, and size is the most critical issue. It was also observed that there were statistically significant differences in the proportion of errors among color, shape, size, and spatial orientation.

## Introduction

With the advancement of the internet, data storage, and search methods, an increasing number of scientific publications and academic papers are being deposited online. This facilitates readers’ access to scientific knowledge and publications. To help in quick and efficient browsing, online resources allow full papers to be stored in several formats such as text, photographs, tables, and charts. This proprietary database helps the reader find it faster, while it raises some problems in understanding data. When tables and charts are positioned closest to their descriptions, the author’s intention will be delivered more clearly. However, it is not surprising that we see so many tables and charts that do not themselves contain information to convey. When a reader fails to read the description, the author loses the ability to communicate his or her information. As a result, the design, arrangement, and organization of data in tables and charts play an important role in information dissemination.

Visualization has long been used as an effective method for transcribing data into information and help people carry out data analysis tasks and make decisions. A number of visual encodings (such as color, shape, and size) can be used individually or together for representing various visualization tasks [1–3]. Misinformation in data visualization has become one of the main issues to convey knowledge more effectively [4, 5]. As opposed to creating effective visualization, misinformation in data visualization receives less attention and becoming one of the major issues for conveying information [5]. Misinformation can be classified into two categories including intentional misinformation and unintentional misinformation [6]. Intentional misinformation [7] in data visualization refers to the use of charts, graphics to distort, hide, or fabricate data in an attempt to deceive users. On the other hand, unintentional misinformation [8] implies providing false and inaccurate information to the end-users because of human cognitive bias and carelessness in designing/selecting visual channels to encode the corresponded data. From the creator’s point of view, the former is controllable while the latter requires a lot of training. This work will be focusing more on the latter one as it is encouraged to build trust in visualization rather than lies [9].

A good visualization tool supports users to get insights (i.e., trends, patterns, and outliers) and extract meaningful information from data. Wilkinson [10] describes the building blocks for constructing a wide range of statistical graphics in his notable book “The Grammar of Graphics”. On the other hand, many articles on the web provide bad examples of visual designs leading to misinterpretation but they are usually viewed from different perspectives. In line with this research direction, Bresciani and Eppler [6] provided a comprehensive review on common errors in data visualization and classified these pitfalls into three categories: cognitive, emotional, and social. Their high-level abstract of the theoretically grounded classification can be used as a checklist for practitioners to examine the visualization for avoiding common drawbacks. However, it is challenging to go through the entire checklist due to the different human cognitive and one’s preference styles. As such, the authors suggested that a more rigorous study should be conducted to “rank the pitfalls according to how common or severe they are.” In response to these needs, in this paper, we try to compromise the most common errors into a structure based on the units of visualization. In another word, we address the following research questions: (R1) What are the most common effects resulting from visualization pitfalls? (R2) What are the most common errors in constructing a representation in terms of color, shape, size, and orientation? Or (R3) Is there any difference in the proportions of common errors among color, shape, size, and spatial orientation? We believe that going from these units would benefit practitioners, especially novice users when they are mostly working with these elements. We expect that the result of this study will be served as a reference for avoiding visualization pitfalls in general. Ultimately, in the long run, we will attempt to answer the questions such as what color [11] should we use to represent data? what visualization types [12, 13] should be taking into account for analysis? what type of scale should we apply? As such, this is preliminary research work to accomplish the goals.

This study is a continuation of a previously published work at the symposium on visual information communication and interaction [14]. The new version was extended by expanding the existing database (Google Scholars) with Scopus, incorporating a text mining technique to extract practical insights from common visualization pitfalls, and examining if there is any difference in the proportions of common errors among color, shape, size, and spatial orientation with Cochran’s Q test and McNemar’s test.

The rest of this paper is structured as follows: First, a literature review relevant to our work was presented. Then, the instruments and processes to conduct the research were provided along with the analytics. After that, the results were presented. Finally, the findings were discussed and concluded.

## Related work

Many recent works have addressed the issues of misinformation in data visualization. Huff [15] showed a variety of ways a designer can trick people, e.g., truncating certain portions of the graph. As such, the discrepancy is more obvious when seen as a whole. This goal can be accomplished by three common strategies [9, 16]: (1) not revealing any data, (2) showing the data incorrectly, and (3) obfuscating the data.

Many authors attempted to provide designers with a boilerplate for creating a successful visualization, ranging from individual graph elements such as colors [17–19] and forms (or chart types) [20] to a detailed data visualization design [21, 22]. However, bad visualization design persists in the presence of guidance, which leads to misinformation that can contribute to considerable problems, especially where individuals heavily rely on the data at hand when making business decisions. Study results raise concerns: what are the possible causes of disinformation visualization? This research is critical in developing a visualization platform that not only meets end-user needs but also mitigates the issues from misinformation and disinformation.

The pitfalls of visualization have been examined in a variety of fields such as information visualization [23–25] - psychological/aesthetic restrictions presented graphic format are highlighted, diagrammatic representations [13, 26–28] using the diagram to understand concept or idea instead of algebraic means, human-computer interaction [21, 29] - potential drawbacks of interactive visualizations, statistical graphic representations [22, 30] - bad examples of representing data visually. The most comprehensive research was provided by Bresciani and Eppler [6], where the authors explored the potential drawbacks in graphic depictions. The authors compiled a list of 51 pitfalls of visualization drawbacks based on interviews with seven experts and divided them into three categories: cognitive, emotional, and social effects.

Unlike the previous research relying on high-level abstractions to avoid visualization errors, we focus on the basic elements that constitute visualization mapping. These elements are color, shape, size, and spatial orientation presented by Munzner [22]. According to Bresciani and Eppler’s research [6], there is a need to review a selection of images to assess the frequency and severity of the flaws. In this study, we aim to alleviate the problems of misinformation in data visualization.

## Methods

### Data sources

For the research, the preferred reporting items for systematic reviews and meta-analyses (PRISMA [31]) model served as a guideline. It is a bare-bones set of evidence-based features meant to help authors report on a wide range of systematic reviews and meta-analyses. Google Scholar and Scopus indexing databases were used to gather data for our study due to their popularity and credible results. The search terms were “misinformation visualization”, “misleading visualization”, “disinformation visualization”, “visualization pitfalls”, “bad visualization design”. Google Image search was used to collect representative images that illustrate visualization pitfalls to avoid direct criticism of previous studies. Table 1 provides the number of papers collected from the two indexing databases according to their keywords.

**Table 1:** Data collection results from Google Scholar and Scopus indexing databases

Keywords	Scopus	Google Scholar
Misinformation visualization	2	41
Misleading visualization	156	306
Disinformation visualization	25	7
Visualization pitfalls	38	665
Bad visualization design	8	94
Total	229	1113

### Data preprocessing

**Fig. 1:**
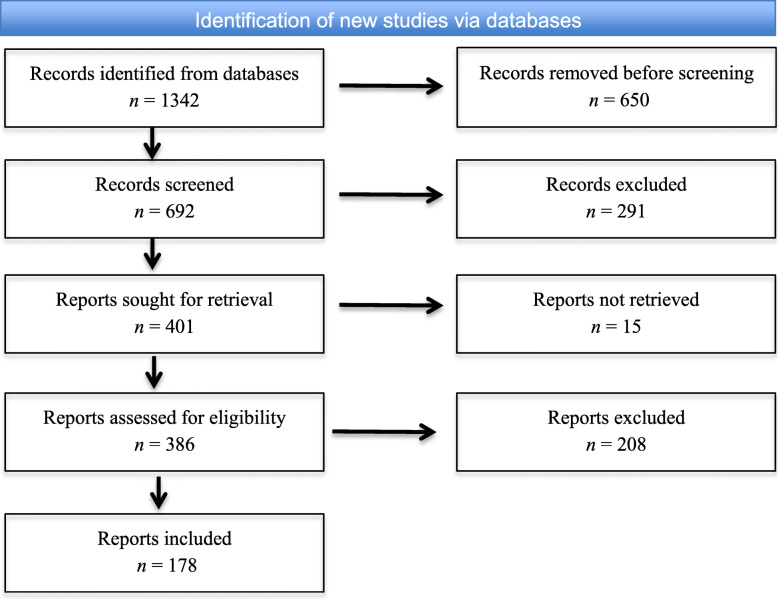
The PRISMA flow diagram

Search results from the database were consolidated into a single excel file format, and duplicated items were removed. The final list of items was filtered by the scientific articles’ abstracts and main contents. Here, we did not exclude items by their titles. Rather, we examined the details of abstracts and main contents to see where the keywords were positioned. It is noted that some of the collected papers contained keywords but were not relevant to our research goal, e.g., some papers did not contain figures to demonstrate the searching keywords. Such papers were excluded from our analysis. In the end, 178 papers were included in the study. Figure 1 depicts the flow of information through the different phases of the systematic review utilizing the PRISMA approach.

### Data extraction

We extracted two types of data from the research papers: figures and their associated descriptions. Each figure is classified into one of four categories (i.e., color, shape, size, and spatial orientation) which indicates a type of violation in visualization. The relevant papers were screened through three phases: (1) checking the title, (2) reviewing the abstract, and (3) scrutinizing the full text of the paper.

### Data analysis

For the first research question, we employed text mining techniques to gain practical insights from the collected data, including association rule mining to find out the relationships among the descriptions of figures. The inputs for this technique were taken from associated descriptions of each figure. We created a word cloud with the most often used words in related explanations, removing any stop words, non-ASCII characters, white space, and duplication. Word clouds are graphical representations of word frequency that highlight terms that appear regularly in a source document. The bigger the term in the visual, the more popular the word was in the text. This visualization method can help evaluators with exploratory textual research by highlighting terms that appear often in a series of interviews, records, or other languages. It may also be used to communicate the most important points or topics during the reporting stage. Association rule mining is a rule-based machine learning technique that seeks out interesting hidden relationships among variables in a dataset. Association rules have been used in a variety of applications, including opinion mining, intrusion prevention, continuous processing, and bioinformatics.

**Fig. 2:**
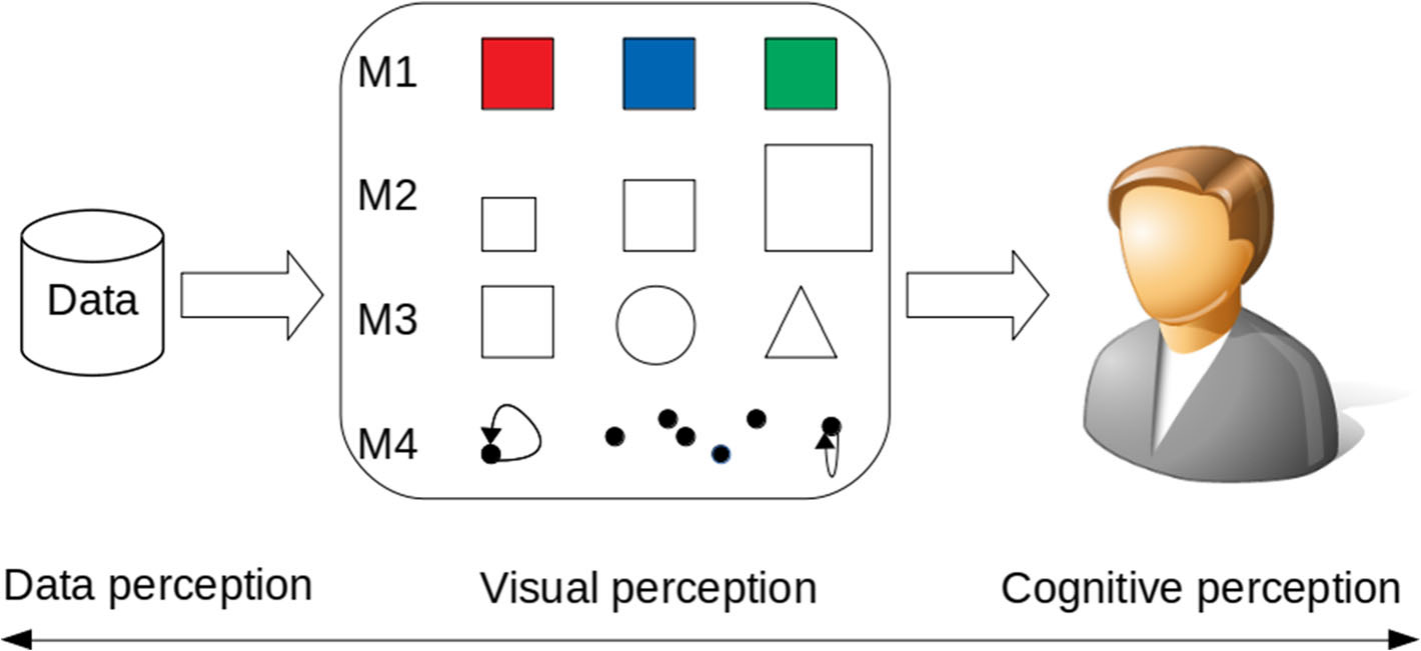
A visual approach for categorizing misinformation data visualization: M1 (color), M2 (size), M3 (shape), and M4 (spatial orientation)

For the second research question, we grouped typical visualization pitfalls into four major categories denoted from M1 to M4 based on the basic elements (color, size, shape, and spatial orientation), as shown in Fig. 2, where “red, blue, and green” represent the color, scaled squares represent the size, “circle, rectangle, and triangle” represent the shape, and scatter black dots represent the spatial orientation. As we move from left to right, misinformation for visual mapping can result from data itself, such as missing data or poor data, to cognitive perception, where color, shape, and scale are perfectly used but misinformation occurs due to cognitive mechanisms. Visual perception is the primary subject of this paper (for more information on data perception and cognitive perception, see Bresciani and Eppler’s study [6]).

For the third research question, we conducted Cochran’s Q test and McNemar’s test [32] to examine if there is any difference in the proportions of common errors among the fundamental units of color, shape, size, and spatial orientation. We examined each paper individually and extracted figures that were violated in terms of any of the four categories. It is noted that only figures generated from data by authors were included for the analysis. Figures taken from photos or other sources were excluded. We encoded “1” as being violated and “0” for not being violated. Thus, our data consists of four binary variables. Statistical analysis was performed with SPSS Version 25.

## Results

### RQ1: What are the most common effects resulting from visualization pitfalls?

**Fig. 3:**
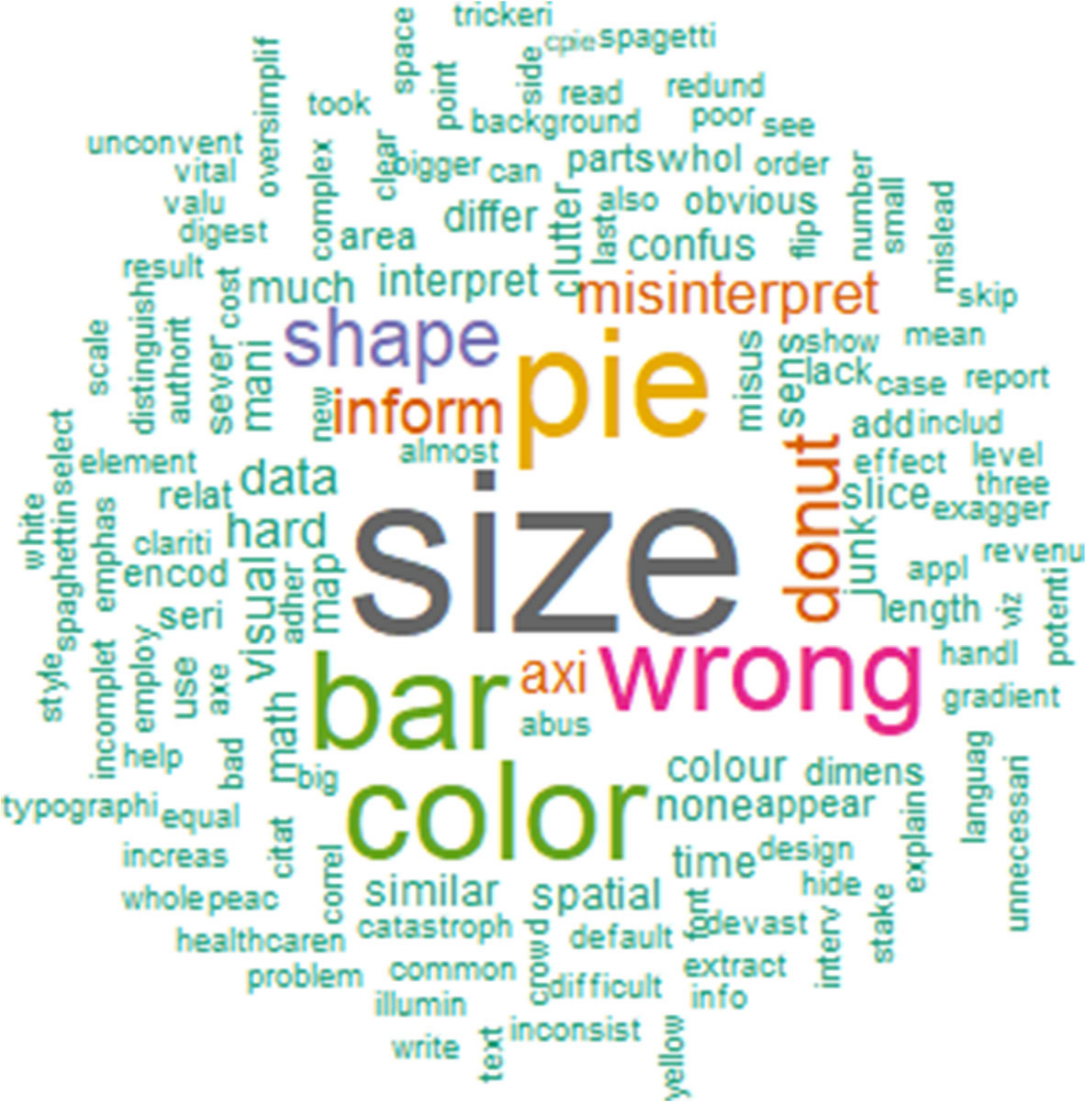
Word cloud constructed from associated descriptions about the figure that encounters visualization pitfalls

After removing special characters in the associated descriptions of figures, we had 363 items with 139 unique words. The items are the remaining words/keywords extracted from the descriptions after cleaning data. Figure 3 depicts the most frequently used terms for each input figure that encounter visualization pitfalls. Frequently used terms in Fig. 3 are shown in larger font sizes in the word cloud. In terms of dominant keywords, besides our proposed categories such as color (72), shape (36), size (132), a majority of misleading visualizations comes from pie chart (83) and bar chart (72), followed by using the wrong chart (63) that incur misinterpretation (21). In addition to the pie chart, donut (33) is another interesting finding that contributes to common errors, it seems that creators are interested in using circles to explain their messages. It is noticed that the terms “inform and axis” are also highlighted in our visual layout which indicates that creators and viewers have to take care of the axis while interpreting data to inform decision-making. We can see other terms that appear to be slightly different from others such as data, hard, and clutter, but they do not stand out apparently.

**Fig. 4:**
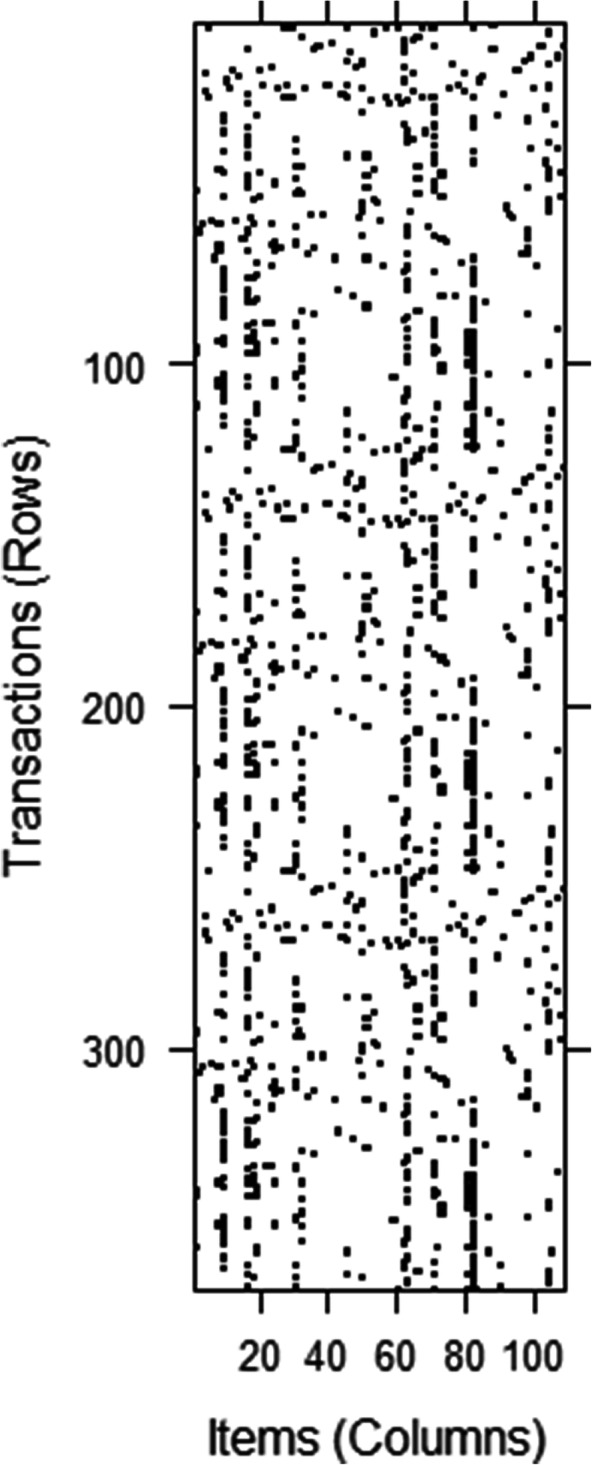
Transactions and items in a matrix

Figure 4 depicts the graphical layout of our transactions and items in the dataset, where black cells denote non-zero elements and empty cells represent zero elements. Rows (transactions) are associated description index of figures, while columns (items) are terms extracted from their corresponding descriptions. In total, we had 370 descriptions and 109 terms. The percentage of non-zero cells in this 370 × 109 matrix is shown by density. It is worth noting that a matrix is considered sparse if most of its elements are empty. On the other hand, if most of the elements are non-zero, the matrix is called dense. The sparsity of the matrix is defined as the number of zero-valued elements divided by the total number of elements. Our dataset showed a density of 0.0312 which means that on average, each description contains 3 to 4 keywords/terms. This is due to the data cleaning process, where all stop words were removed.

**Fig. 5:**
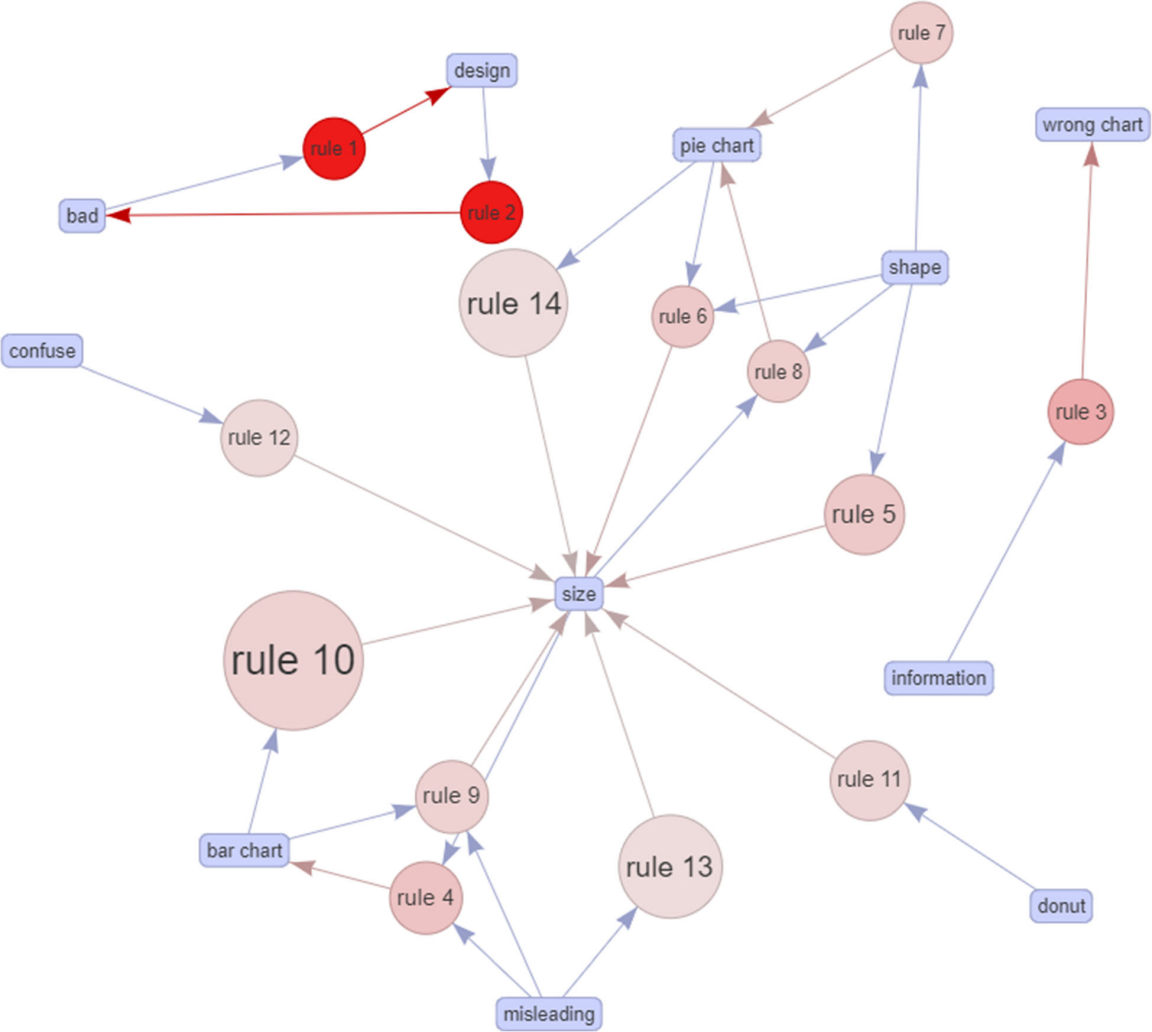
Graph-based visualization on the relationship between individual items in the ruleset

Table 2 showed a list of 14 rules generated from the A-Priori algorithm [33]. This algorithm necessitates both minimal support and a minimum confidence constraint. If no threshold value is defined, the function will consider the default values of support and confidence threshold as 0.1 and 0.8, respectively. Since the elements in the matrix are sparse, support and confidence are modified with lower values (support = 0.04 and confidence = 0.5) to mine the most potential rules rather than using default values. Support is the percentage of transactions (or figures) that contain both words in a rule and confidence is the strength of that rule. The A-Priori algorithm outputs the highest value of support (0.162), confidence (1), and lift (11.012) respectively. The lift value is an indicator of a rule’s significance. If the lift value is greater than 1, it indicates that the rule body (or Rhs) and the rule head (or Lhs) appear together more often than predicted, implying that the occurrence of the rule body has a positive impact on the occurrence of the rule head. On the other hand, if the lift value is less than 1, it implies that the occurrence of the Rhs has a negative effect on the occurrence of the Lhs. Table 2 shows that all lift values are greater than 1, indicating that items in the rules are positively correlated. It is noted that more than half of items (57.14 %) in the rule body involve ‘size’, which means that size is the most critical issue in visualization pitfalls. This statistical data is supported by the visual graph as depicted in Fig. 5, where size is the center of the constructed network with a total of 10 connections from other rules. This network illustrates associations between items and rules. Bigger circles indicate higher support, while the red color implies higher lift. One interesting pattern revealed by the algorithm is when creators use size to express information in a bar chart, then misleading is likely to occur (rule 13, 14). In addition, incorrect usages of size can lead to confusion.

**Table 2:** Association rules mining in the figures’ descriptions

Lhs	Rhs	Support	Confidence	Coverage	Lift	Count
{design}	{bad}	0.041	0.714	0.057	11.012	15
{bad}	{design}	0.041	0.625	0.065	11.012	15
{information}	{wrong chart}	0.049	0.667	0.073	4.836	18
{donut}	{size}	0.056	0.700	0.082	1.837	21
{confuse}	{size}	0.048	0.600	0.082	1.574	18
{shape}	{pie chart}	0.041	0.500	0.082	2.467	15
{shape}	{size}	0.081	1.000	0.082	2.624	30
{misleading}	{size}	0.097	0.522	0.186	1.369	36
{bar chart}	{size}	0.162	0.769	0.212	2.019	60
{pie chart}	{size}	0.105	0.520	0.202	1.364	39
{pie chart, shape}	{size}	0.040	1.000	0.041	2.624	15
{shape, size}	{pie chart}	0.041	0.500	0.081	2.466	15
{bar chart, misleading}	{size}	0.064	0.800	0.082	2.111	24
{misleading, size}	{bar chart}	0.064	0.667	0.097	3.162	24

A-Priori Algorithm was used with a support threshold of 0.04 and a confidence threshold of 0.5

### RQ2: What are the most common errors in constructing a representation in terms of color, shape, size, and orientation?

#### M1: Misinformation due to color violation

**Fig. 6:**
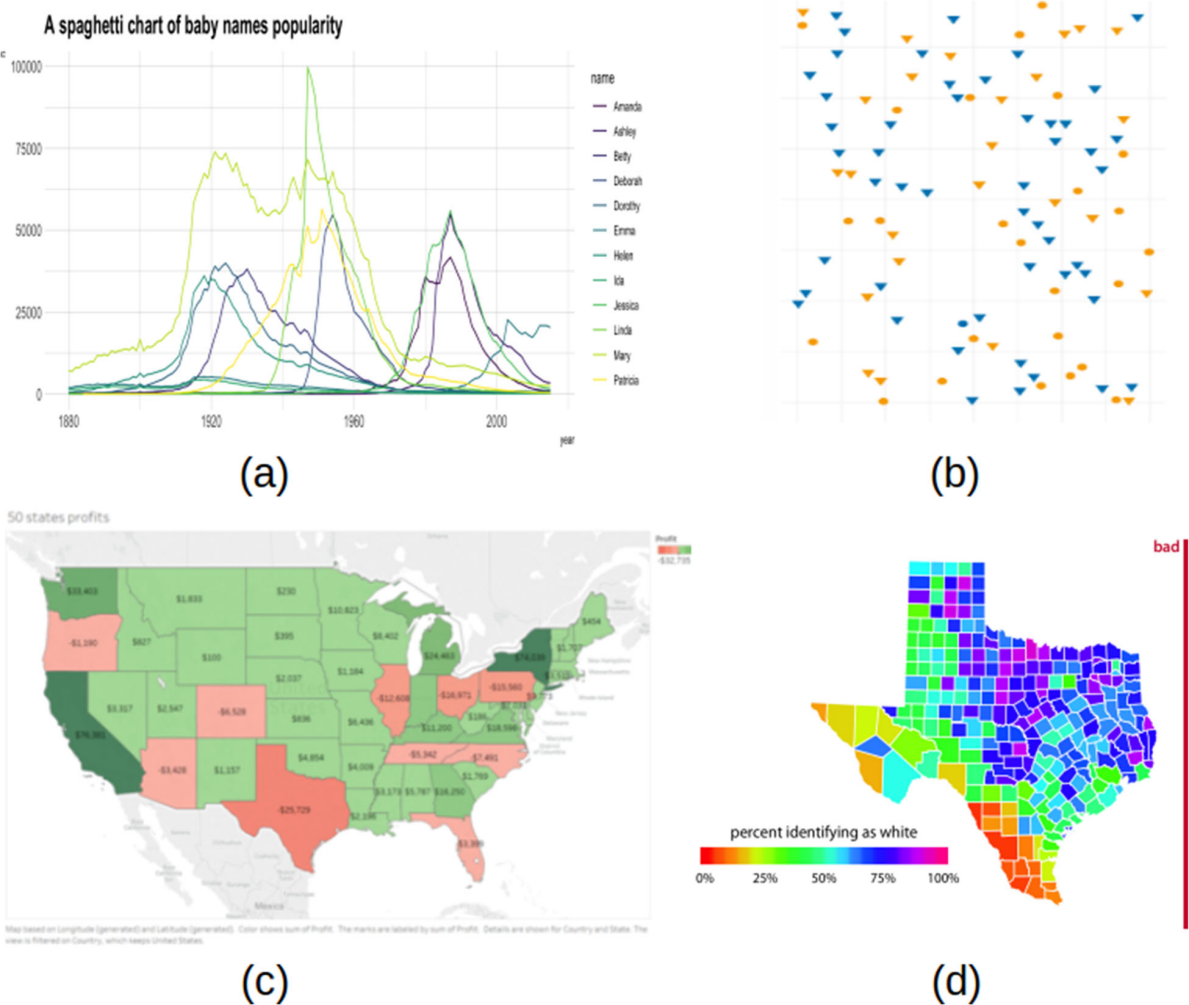
Misinformation due to color violation: **a** using too many colors [36], **b** unnecessary color usage [37], **c** color against the norm [38] and **d** rainbow color scale [39]

As shown in Fig. 6, one of the most common visualization mistakes is the use of too many colors. Healey et al. [34] argued that the human eye can only process five to seven well-chosen colors pre-attentively, and qualitative color scales are thought to work best when three to five different colored categories are present. Choosing a color scheme is another critical issue when different categories are encoded by related color on a spectrum that makes it difficult to distinguish one from another as in Fig. 6a - legend. In some cases, the use of continuous color even gets worse leading to misinterpretation of data [35], especially when using a non-monotonic scale or rainbow scale [11]. It can be seen from Fig. 6d that the colors at the beginning and the end are almost equivalent (circulated color scales). Figure 6b shows using the same color for different categorical data. Although each category can be annotated by a distinct shape, color is often misused to represent an additional dimension especially when the granularity of the new dimension is too low. It takes more time to locate the blue circle point of interest. Another common bad habit is using the color against the norm: it is often found in the practice that light color is usually for less density while darker color is for higher density or green for a healthy indicator vs. red for abnormal value. The example illustrated in Fig. 6c misused red color business gains and green color for business losses. A first glance, this visualization misled us that the economy is flourishing.

#### M2: Misinformation due to shape violation

**Fig. 7:**
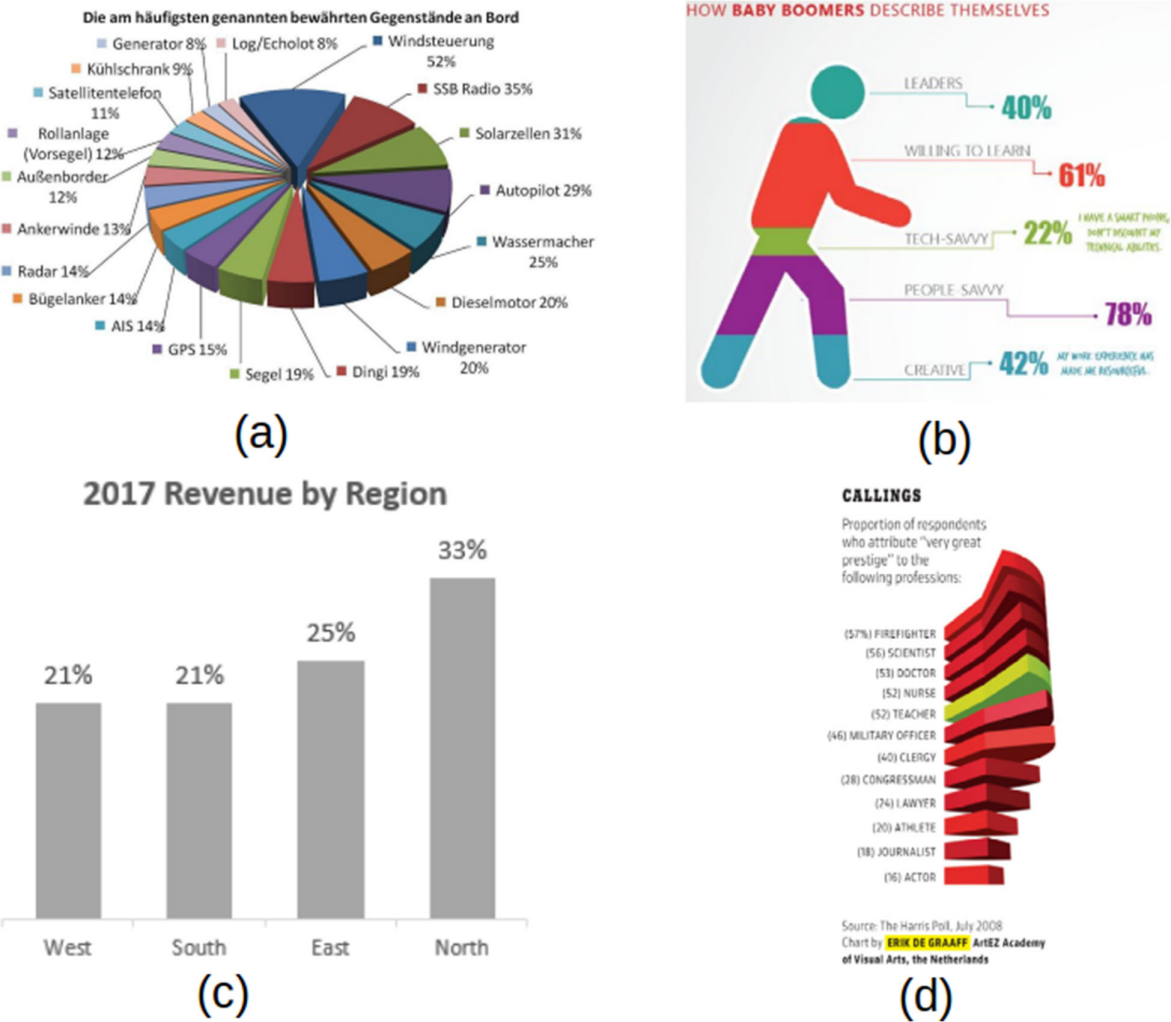
Misinformation due to shape violation: **a** using 3D pie chart for comparing proportion [40], **b** using wrong visualization chart type, **c** pie-chart should be used [41], and **d** bar chart should be used [42]

The shape can be violated due to the use of inappropriate chart types. In fact, this is the most common misleading chart that we found in the literature. Particularly in the case of using a pie chart instead of a bar chart and vice versa. A pie chart is a type of graph in which a circle is broken down into segments (i.e., slices of pie) that each represent a proportion of the whole. Each slice of the pie chart is represented by a percentage value accumulating to 100%. Figure 7a illustrates the improper use of the pie chart where each slice adds up to more than 100%. Even though the original intention was to compare values among different categories, this chart also violates color choices (so many colors, or similar colors for different categorical data). Common practice [37] suggested that the pie chart can be best used with less than seven categories (same magic number as in color) due to the difficulty for the eye to distinguish relativity of size between each segment. In this case, the bar chart should be a better choice. Figure 7c shows an opposite case where the pie chart should be taken into consideration instead of using the bar chart for comparing proportional data as percentages add up to 100%.

The second misleading information when performing visual mapping is to use a shape that does not reflect the information provided. It can be seen from Fig. 7b that the body part misleads users from what it means. Like the case of the pie chart, proportionality does not add up to 100% or areas cannot be compared visually.

Another common misleading information is the use of a high-dimensional chart to represent lower-dimensional data. This is often due to the visual appeal of 3D charts or the priority of prioritizing design and technology over conveying information of the chart as it can be seen from Fig. 7a and d where heights of each slice did not provide any additional information.

While doing visual mapping, the second deceptive information is to use a shape that does not represent the information presented. Figure 7b demonstrates how the body part misleads people on what it means. Proportional, like the pie map, does not add up to 100%, or regions cannot be physically compared. Another example of deceptive evidence is the use of a high-dimensional map to display low-dimensional details. This is often attributed to the aesthetic appeal of 3D charts or the importance of prioritizing architecture and technology over conveying detail of the map, as seen in Fig. 7a and d, where the heights of each slice provided no additional details.

#### M3: Misinformation due to size violation

**Fig. 8:**
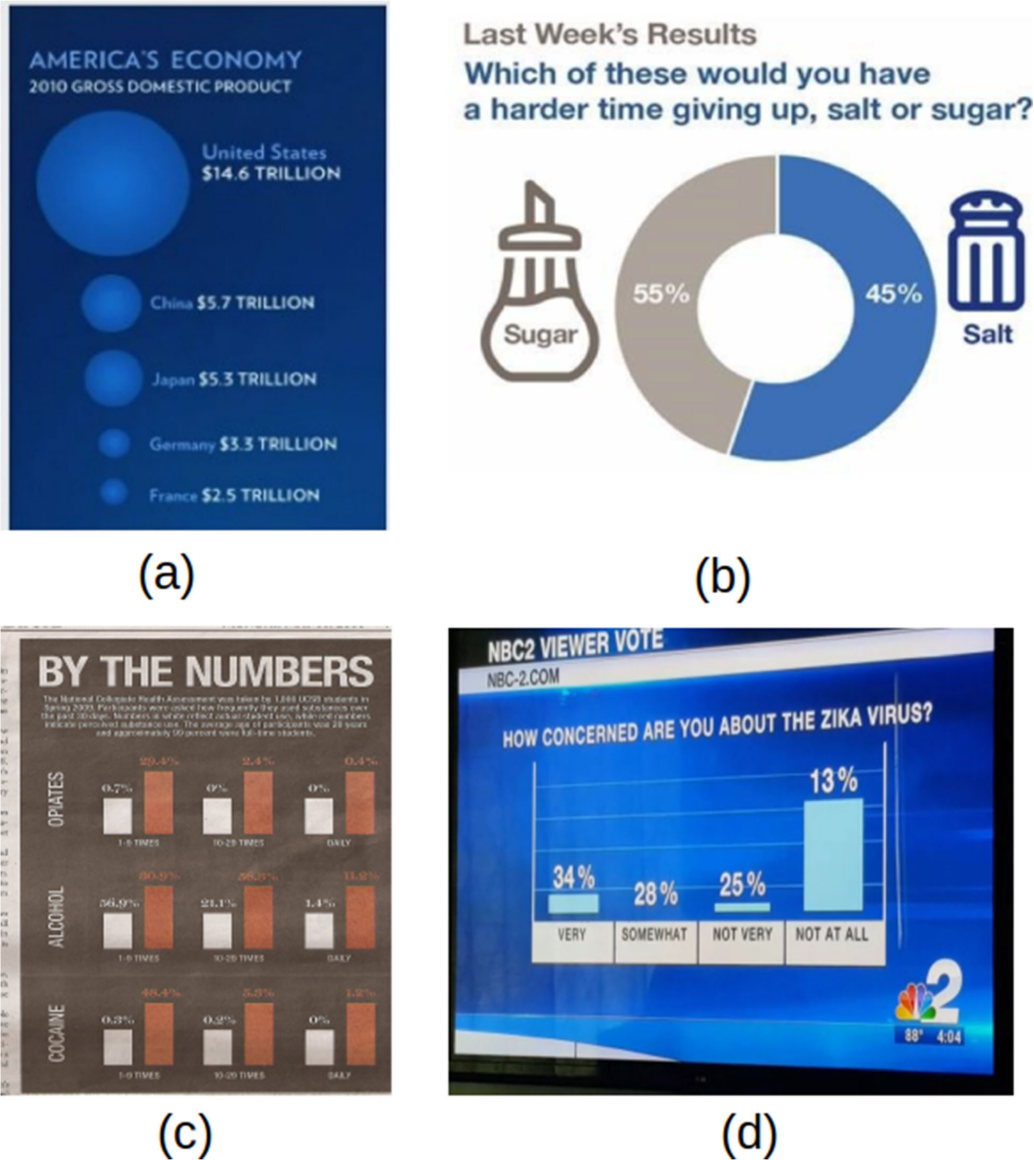
Misinformation due to size violation: **a** using wrong scale [43], **b** size is inverted [40], **c** all sizes are equal [44] and **d** size with no meaning [45]

Regarding size infringement, the most common problem is to use a single size dimension scale to express a two-dimensional value. For example, in Fig. 8a, the diameter was used for the scale to display the proportion to GDP, but users often viewed the chart from the perspective of the region. In this case, the field expands exponentially (i.e., quadratic vs linear). Mathematically, 14.5 trillion is 2.56 times larger than 5.7 trillion, but the region seems to be 6.5 times larger.

Careless designers continue to apply incorrect labeling to visual mapping, especially where the chart is labeled manually without the assistance of automatic graphic software. Figure 8b represented this scenario in which 55 % and 45 % could be swapped. Surprisingly, comparable findings in this form of pitfall are typically seen from news channel writers. Another method of softening the detrimental impact of a section is to use a light color, but we do not mention it in our study because it is considered an intention.

The use of equivalent scale shapes supplemented with numbers for contrast is the second common deceptive graph pitfall that belonged to this group, shown in Fig. 8c. Similarly, to orange bars, all-white bars are the same height. Colors are often misused in three separate categories; however, the most serious flaw is the appearance of height where no meaning is present (0 % value of the white bars in the rightmost figure). These common pitfall patterns can also be seen in a pie chart of equal-sized parts.

Another pitfall is to use a graphic encoding that is unrelated to the values it contains, as seen in Fig. 8d, where the height of each bar chart does not fit the value above it. Did the author use an inverted scale? Then 34 % should be the lowest, and where is the bar chart for the “SOMEWHAT” category? While this form of expression is uncommon in academia, social media has had many instances of it.

#### M4: Misinformation due to spatial orientation violation

In this study, we would use a subset of spatial features including spatial and temporal mapping which engages space and time, respectively.

**Fig. 9:**
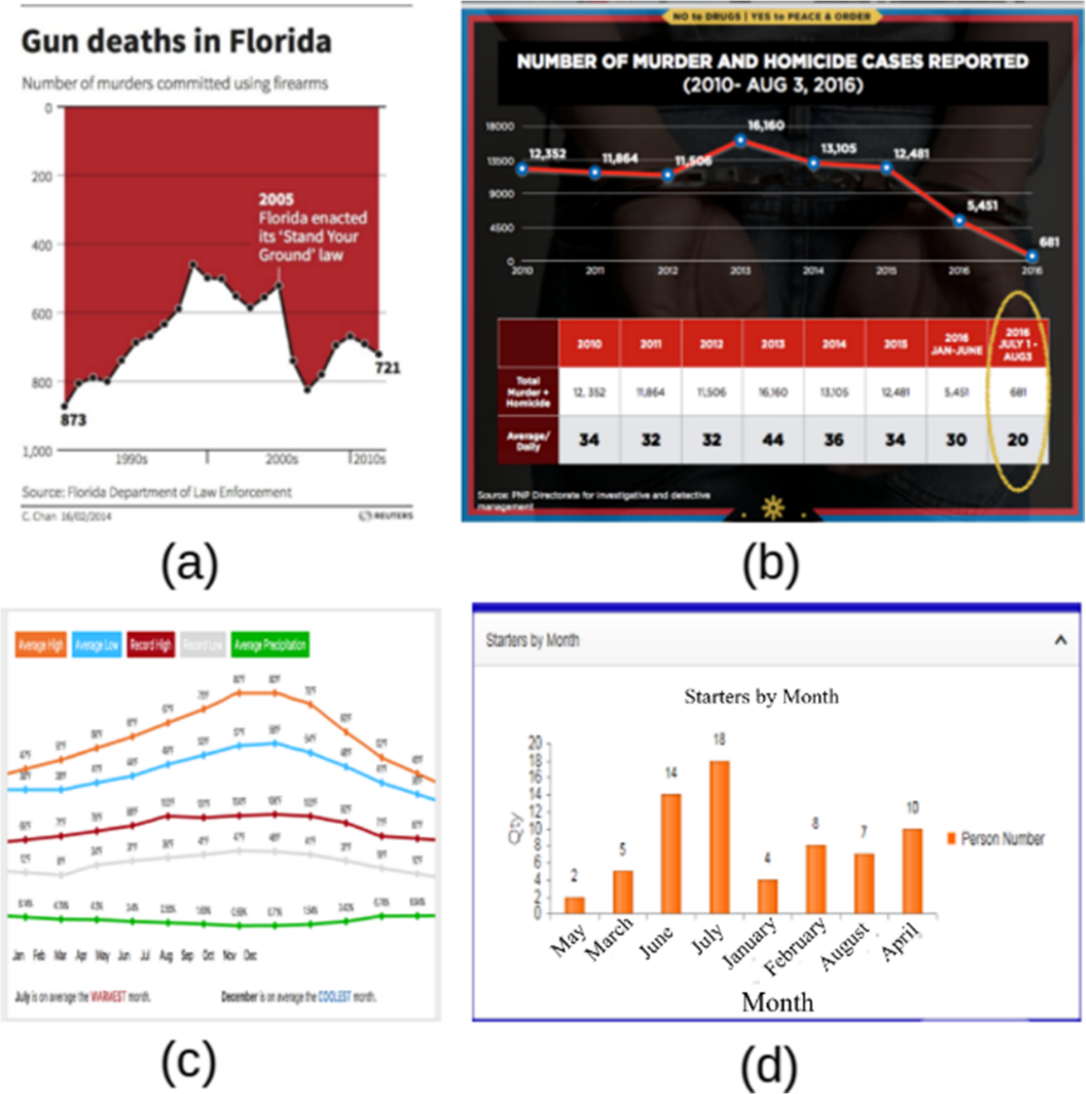
Misinformation due to spatial and temporal violation [46]: **a** inverted axis, **b** data split out, **c** missing axis to line up data, and **d** mixing time data

Inverting the vertical axis is a technique that allows viewers to see the flip side of the original data. In practice, the flip-side data is intentionally removed to convey other information. Figure 9a is the most controversial chart being discussed whether it was created to deceive users [46]. For most viewers, this graph first gave the impression that gun deaths declined sharply after 2005 due to the illusion of the line chart, especially in the presence of black dots. Gun deaths increased. As indicated in ref. [46] “the artist does not appear to have intended the graph to be deceptive. Her view was that deaths are negative things and should be represented as such.” However, the focal point we want to address was that misleading information would make people feel that living in Florida get safer after the incident. A similar story can also be found in Fig. 9b. Omitting data, truncating the axis (or non-zero-base axis), and over zooming to see the difference are popular techniques [9, 16] to make a deceptive graph. From our point of view, in Fig. 9b original data remains but misleading information still exists.

The most common problem with spatial orientation violation is reordering time series results. When creating a graph with a timestamp in one dimension, the order of data points at each time level is shown in Fig. 9d, where months appear to be in a random position. The direction would be totally destroyed if the data are shuffled. Careless charting can also result in the lack of one dimension, shown in Fig. 9c, where the author attempted to combine line graphs from different figures into one. We can see from the contradictory data unit and value location that does not match the upward trend. Misinformation due to data and visual interpretation is most often seen in the form of a table shown in Table 3, where viewers must make at least two attempts to locate the absolute meaning (i.e., compare the length and compare the leftmost values). In this case, the order of magnitude in space is broken.

**Table 3:** Misinformation due to data and cognitive perception

Source sector	Total emissions
Electricity generation	652314
Fires	14520530
Fossil fuel combustion	1499367
Industrial processes	2414055
Miscellaneous	33786
Non road equipment	22414896
On road vehicles	62957908
Residential wood combustion	2704197
Road dust	0
Solvent use	3294
Waste disposal	2018496

### RQ3: Is there any difference in the proportions of common errors among color, shape, size, and spatial orientation?

Table 4 shows that the most frequently observed pitfalls were in size (69.4%). Color and shape showed a similar number of cases (39.7% and 37.2%, respectively). Spatial orientation had the least case (14.9%).

**Table 4:** Frequency table of color, shape, size, and spatial orientation in visualization pitfalls

	Color	Shape	Size	Spatial orientation
Correct (0)	219 (60.3%)	228 (62.8%)	111 (30.6%)	309 (85.1%)
Incorrect (1)	144 (39.7%)	135 (37.2%)	252 (69.4%)	54 (14.9%)

We conducted Cochran’s $Q$ test and McNemar’s test [32] to examine if there is any difference in the proportions of common errors among color, shape, size, and spatial orientation. Cochran’s $Q$ test determined that there was a statistically significant difference in the proportion of errors among the features of color, shape, size, and spatial orientation, $Q$(3) = 207.047, *p* < 0.001. This implies that at least two of the features had a different proportion of errors. Post hoc analysis with McNemar’s test was conducted. Table 5 shows the 2 × 2 classification tables for McNemar’s test. There were no significant differences between color and shape (*χ*^2^ = 0.547, *p* = 0.460). However, there was a statistically significant difference in proportions of errors between color and size (*χ*^2^ = 54.519, *p* < 0.001), color and spatial orientation (*χ*^2^ = 42.586, *p* < 0.001), shape and size (*χ*^2^ = 84.629, *p* < 0.001), and between size and spatial orientation (*χ*^2^ = 132.003, *p* < 0.001). The findings indicate that size is most frequent in the common pitfalls, which is statistically different in frequency from other features. Color and shape are the second most frequent common errors, which are statistically different from spatial orientation in terms of frequency. However, there is no significant difference between color and shape.

**Table 5:** 2 × 2 classification tables for McNemar analysis

		Shape	
		0	1
Color	0	165	54
	1	63	81
		Size	
		0	1
Color	0	60	159
	1	51	93
		Spatial orientation	
		0	1
Color	0	171	48
	1	138	6
		Size	
		0	1
Shape	0	90	138
	1	21	114
		Spatial orientation	
		0	1
Size	0	63	48
	1	246	6

## Discussion

It is undeniable that visualization is becoming more common, especially in the age of the Internet of Things, where millions of data points are created every day. To succeed, we need to provide a big picture in a succinct graphic style to help customers digest details faster. When we screened the papers for the study, we discovered that the majority of the graphical layouts were created by hand or produced by a program using built-in features. Only a few studies used specialized software to present results. Part of the problem stems from developers’ lack of programming expertise, which forces them to rely on free software. In some cases, these tools have a fine, suggested interface (e.g., Google Form automatically generated charts based on survey data), but in others, the layout is much worse than if it were created manually (e.g., Google Form only generates one type of chart for numerical data without considering the number of categories). As a result, developers would use these preset visualizations without regard for visual interface clarity. Another risk that can trigger visualization pitfalls stems from the writing paper guidelines. Although there is a straightforward, concise description of how to write and section (e.g., abstract, introduction, methodology, and conclusion), there is no guidance on how to correctly present data in figures. As a result, authors place a greater emphasis on content rather than visual presentation. This problem is addressed in the area of data visualization, where instructions for presenting data can be found in the paper’s template. However, these recommendations are not systematic, making a boilerplate graphic suggestion for both inside and across fields challenging. We expect that artificial intelligence will be modified in the coming years to automatically screen and identify possible common errors based on research questions and the evidence presented. As a result, developers will be able to concentrate on mining the content rather than thinking about deceptive interfaces.

## Conclusions

This paper dealt with typical visual representation pitfalls. We gathered data from two indexing databases, pooled it, and grouped it into four groups based on data representation units. The most popular visualization pitfalls from each segment were extracted, and data in each section was analyzed using both qualitative and quantitative methods. Word frequency from Word Cloud visualization shows that size is the most dominant keyword found in the descriptions of figures, followed by pie, bar charts, and color. Association rule mining reported that size is the center of the constructed network with a total of 10 connections from other rules and it plays a major concern in visualization pitfalls. Cochran’s Q test and McNemar’s test showed that there was a statistically significant difference in the proportion of errors among the fundamental units of color, shape, size, and spatial orientation. The size issue was most frequent in common pitfalls, followed by color and shape. Spatial orientation had the least case. This paper’s contribution can be thought of as a fine-grained (or subset) of general visualization pitfalls, with a focus on visual perception. We hoped that our findings would aid in the creation of a taxonomy of common errors at the information stage. User interface analysis on visual interpretation will be undertaken in the future with a more detailed test design, taking into account the user’s cognitive loads and cognitive style.

## Data Availability

The datasets used and/or analyzed during the current study are available from the corresponding author on reasonable request.

## Abbreviations

PRISMA: Preferred reporting items for systematic reviews and meta-analyses
